# The impact of grey zones on the accuracy of agreement measures for ordinal tables

**DOI:** 10.1186/s12874-021-01248-3

**Published:** 2021-04-14

**Authors:** Quoc Duyet Tran, Anil Dolgun, Haydar Demirhan

**Affiliations:** 1 An Giang University, VNU-HCM, Long Xuyen City, An Giang Province, 076 Vietnam; 2 Mathematical Sciences, School of Science, RMIT University, Melbourne, Victoria, 3000 Australia

**Keywords:** Grading variation, Inter-rater agreement, Kappa coefficient, Monte Carlo simulation, Reliability, Weight, Table generation

## Abstract

**Background:**

In an inter-rater agreement study, if two raters tend to rate considering different aspects of the subject of interest or have different experience levels, a grey zone occurs among the levels of a square contingency table showing the inter-rater agreement. These grey zones distort the degree of agreement between raters and negatively impact the decisions based on the inter-rater agreement tables. In this sense, it is important to know how the existence of a grey zone impacts the inter-rater agreement coefficients to choose the most reliable agreement coefficient against the grey zones to reach out with more reliable decisions.

**Methods:**

In this article, we propose two approaches to create grey zones in simulations setting and conduct an extensive Monte Carlo simulation study to figure out the impact of having grey zones on the weighted inter-rater agreement measures for ordinal tables over a comprehensive simulation space.

**Results:**

The weighted inter-rater agreement coefficients are not reliable against the existence of grey zones. Increasing sample size and the number of categories in the agreement table decreases the accuracy of weighted inter-rater agreement measures when there is a grey zone. When the degree of agreement between the raters is high, the agreement measures are not significantly impacted by the existence of grey zones. However, if there is a medium to low degree of inter-rater agreement, all the weighted coefficients are more or less impacted.

**Conclusions:**

It is observed in this study that the existence of grey zones has a significant negative impact on the accuracy of agreement measures especially for a low degree of true agreement and high sample and tables sizes. In general, Gwet’s AC2 and Brennan-Prediger’s *κ* with quadratic or ordinal weights are reliable against the grey zones.

**Supplementary Information:**

The online version contains supplementary material available at (10.1186/s12874-021-01248-3).

## Background

Agreement studies are important in measuring the degree to which multiple specialists, reviewers, examiners, or raters agree on an issue. The issue of interest can be the severity of a disease, classification of a biological specimen in terms of its pathological grade, or an assessment of the degree of learning over some test results. The degree of inter-rater agreement depends on the individual raters [1, 2]. There can be biases independent of a characteristic that is assessed by the raters such as gender-related biases [2]. Different subjective evaluations due to different reasons may influence the assessment. Such biases introduce a variation into the decision making process in practice. This is a widely discussed issue especially in the medical and veterinary practice [3–6]. In pathology, some protocols have been developed to standardize the approaches of pathologists to grade the tumors and report the results to mitigate this variation [5, 6]. For instance, [6] report a high variation in the histologic grading of mast cell tumors of dogs due to the use of different references describing grading systems. Recently, van Dooijeweert et al. [7] report a substantial variation in histologic grading of ductal carcinoma in situ of the breast among pathology laboratories and between pathologists within laboratories in Netherlands. The reason of the variation is hypothesised to be the use of non-uniform guidelines for grading. As seen in this instance, the subjective perception of raters even within the same laboratory would be different in grading tumors. There would also be other causes of variations. In the assessment of the grade of a disease, based on their level of experience, one of the raters would tend to assign higher grades to be on the safe side while the other rater tends to grade lower. For instance, Zbären [8] reports that when the frozen section samples are assessed by experienced pathologists in analyzing frozen section of salivary gland neoplasms, tumor typing and grading have a notably high accuracy. When it is translated into the agreement studies, due to any of such variations, the distinction between the categories becomes not as sharp as for all raters, resulting in the existence of grey zones. So, for example, an agreement table composed of pathologists’ assessments of the same patients may have grey zones because of following different references for grading or not having similar levels of experience. Generally, a grey zone occurs in inter-rater agreement studies when the raters cannot/do not clearly distinguish any pair of adjacent categories in the contingency table composed of ordinal ratings. The reasons for having a grey zone is as diverse as the reasons of variations seen in pathology or other similar areas. It should be noted that we assume that there is no misclassifications due to the human error. When there is a grey zone in the table, raters tend to fall more in one category, in general, preferring the mid-category, and this leads to an artificial increase in the disagreement between two raters.

In the medical literature, the existence of grey zones is reported in a different context, namely quantitative diagnostic testing, where it depends on the characteristics of respondents rather than the deviations from a balanced distribution of cell counts in the upper and lower triangles of a contingency table due to the rating behaviour of assessors, which we call “table structure” throughout the manuscript. The existence of a grey zone in quantitative testing can impact the discriminatory performance of a diagnostic test [9]. In this context, recently, Kossaify [10], and Draper et al. [11] worked on the application of the grey zone approach in the estimation of the range of cut-points values for the diagnosis of heart failure. Kossaify [10] explained the concept of the grey zone in medicine as a fact including a daily controversy in medical practice. Pereira et al. [12, 13], and Mazzu-Nascimento et al. [14] applied the grey zone approach in the context of screening immunoassay. Mazzu-Nascimento et al. [14] explained a grey zone as an area with a categorized degree of uncertainty to provide a more reliable measure. These studies mainly focus on dealing with or mitigating the impact of grey zones caused by the subjects and/or the diagnostic tools. The approaches to mitigate the impact of grey zones include trying to narrow down the grey zone [10] or partitioning the scale used for diagnosis by adding one more category between well-known categories based on the hesitation to choose one of them [9, 15]. However, studies focus on the grey zones created by assessors, who rate the same subjects into ordinal levels, such as the grade of a disease, are quite limited. In this study, we consider the case where the personal judgements of the assessors are influential on the existence of a grey zone rather than the characteristics of subjects related to a diagnosis. Thus, the impact of grey zones on the structure of an agreement table; hence, on the inter-rater agreement measures and the reliability of the agreement measures against the grey zones are of interest in this study.

The accuracy of the inter-rater agreement measures is expected to be impacted by the existence of grey zones in the ratings of one or both raters. Thus, it is crucial to understand the impact of grey zones in the inter-rater agreement context when combined with other factors such as the sample size, the structure of the table, the number of categories, and the degree of agreement. Although there are studies on the accuracy of different agreement coefficients in general [16] or on particular concepts [17], the impact of grey zones on the agreement measures has not been thoroughly studied yet. Choosing the most reliable inter-rater agreement measure against the existence of grey zones based on the results of our study has a high potential to better inform medical decision making. Although there are studies in the literature of inter-rater agreement context suggesting that marginal distributions of raters have an impact on the inter-rater agreement, to the best of our knowledge, they never associate “the difference in marginal distributions” to a “grey zone effect” as we deal with in this research. In this paper, a definition of the grey zone is given within the inter-rater agreement context and its impact on the accuracy of inter-rater agreement measures is studied through extensive simulations. Then, the accuracy and reliability of the weighted inter-rater agreement measures for ordinal tables are assessed to recommend the measures that can be used under specific circumstances.

Overall, the accuracy of the inter-rater agreement measurements for ordinal data is assessed when there is a grey zone for one of the raters. Varying sample sizes, table structures, type of the agreement measurements, weighting schemes, the value of the true inter-rater agreement, and the number of categories are taken into consideration. To the best of our knowledge, this research is the first study that proposes a method to create a grey zone in a given agreement table for simulation purposes. This provides researchers with a method to simulate grey zones in other contexts as well. Since the way a grey zone impacts the accuracy of the inter-rater agreement for ordinal tables is fully explored with an extensive simulation study, another contribution of this study is to show the impact of grey zones on the accuracy of inter-rater agreement measures when combined with other factors such as sample sizes, table structures, number of categories, levels of agreement, agreement measures, and weights.

### Motivating example: diagnostic classification of films for breast cancer

Boyd et al. [18] reported the ratings of two radiologists on the diagnostic classification of films for breast cancer. The xeromammograms were chosen by two radiologists from two different Breast Cancer Detection Demonstration Project screening centers. The films from each patient included a mediolateral and craniocaudal figure of each breast. For patients with more than four years after taking the film and never commit to breast cancer were coded as “normal”. One radiologist took films thought to be illustrative of the three primary diagnostic instructions; the other radiologist chose graphics from the Department’s instructing record that for tumours indicated just unobtrusive radiologic highlights of the ailment and for the benign abnormalities confirmed shared radiologic symptoms with the minimal breast cancers. In this example, pathologists refer to different references to grade the films similar to the cases reported by Northrup et al. [6] and and van Dooijeweert et al. [7]. The agreement table presented in Table 1 shows the diagnostic classification of 85 films according to two radiologists. Naturally, the benign abnormalities illustrated some radiological signs that are similar to the minimal breast cancers (coded as “Suspected cancer”). From the unbalanced distribution of the cell counts around the main diagonal of the table as well as the marginal counts, we infer that Radiologist B attempted to select more in category “Benign disease” (38) than in “Suspected cancer” (16) while Radiologist A classifies more in “Suspected cancer” (29) than “Benign disease” (22). This causes inflation in the count of controversial class (“Suspected cancer”, “Benign disease”) to 9 while that of (“Benign disease”, “Suspected cancer”) is 1.

**Table 1:** Diagnostic classification of films

		Radiologist B				
	Diagnosis	Normal	Benign disease	Suspected cancer	Cancer	Total
**Radiologist A**	Normal	21	12	0	0	33
	Benign disease	4	17	**1**	0	**22**
	Suspected cancer	3	**9**	15	2	**29**
	Cancer	0	0	0	1	1
	Total	28	**38**	**16**	3	85

The counts related to the grey zone are in bold

For patients who had films with benign breast disease, Boyd et al. [18] mentioned that the interpretation of these signs can be different among radiologists. Practically, these patients are required to check biopsy because their radiologic symptoms cannot be dependably recognized from cancer. This finding may emerge in portion from subjective characteristics such as diagnostic habits shaped in clinical hone, where there is an excessive prevalence of most cancers within the films inspected, and particular significance is set on the need to prevent losing patients with breast cancer. This would be the reason why the Radiologist A tends to place more results in “Suspected cancer” while fewer results in “Benign disease” to make a safer diagnosis. The grey zone includes both “Benign disease” and “Suspected cancer.” As a potential impact of the grey zone, Radiologist A prefers to “Suspected cancer” category, (around 34% for “Suspected cancer” category and 26% for “Benign disease” category), whereas the Radiologist B tends to choose the “Benign disease” category (approximately 45% for “Benign disease” category and 19% for “Suspected cancer” category).

It is not possible to know the actual cell counts on Table 1 in the case that Radiologist A does not tend to make safer diagnoses (i.e. tendency to select suspected cancer category when hesitated). However, some hypothetical scenarios for the same table can be generated to demonstrate the impact of the grey zone on the inter-rater agreement. In these scenarios, we change the associated cell counts to remove the existing impact of grey zones in the data. Then, we compare the corresponding inter-rater agreement measures to infer the impact of the grey zone.

Table 2 displays three hypothetical scenarios for Table 1. With this table, we demonstrate how the frequencies in Table 1 would look like if Radiologist A doesn’t have any tendency to rate more on the “Suspected cancer” category rather than the “Benign disease” category. Specifically, if there was no grey zone for Radiologist A in Table 1, they would place more ratings in benign category (hence the row total for the benign category would be more than originally observed) and would place fewer ratings in the “Suspected cancer” category (hence the row total for the suspected cancer category would be less than originally observed).

- In scenario 1, we fix the marginals for Radiologist B and move the majority of Radiologist A’s suspected cancer ratings (which are on the main diagonal) to the benign disease category. Thus, the agreement is increased for “Benign disease” category and reduced for “Suspected cancer” category. The hypothetical cell counts under this scenario are represented with *†* symbol in Table 2.

**Table 2:** Hypothetical scenarios for Table 1

		Radiologist B				
	Diagnosis	Normal	Benign disease	Suspected cancer	Cancer	Total
**Radiologist A**	Normal	21	12	0	0	33
	Benign disease	4	17, 26 ^*†*^, 26 ^*‡*^, 26 ^*§*^	1, 14 ^*†*^,1^*‡*^, 3 ^*§*^	0	22, 35 ^*†*^,31^*‡*^,33^*§*^
	Suspected cancer	3	9, 0 ^*†*^, 0 ^*‡*^,0^*§*^	15, 2 ^*†*^,15^*‡*^,12^*§*^	2	29, 16 ^*†*^,20^*‡*^,18^*§*^
	Cancer	0	0	0	1	1
	Total	28	38	16	3	85

*†*: Scenario 1; *‡*: Scenario 2; *§*: Scenario 3.

Only the marked cells are changed to create three hypothetical scenarios
- In scenario 2, we fix the marginals for Radiologist B and move all of the Radiologist A’s suspected cancer ratings to the benign disease category on the main diagonal. So, the agreement is increased for “Benign disease” category. The hypothetical cell counts under this scenario are represented with *‡* symbol in Table 2.
- In scenario 3, in addition to the movement of frequencies described in scenario 2, we move only 2 of the cases that are on the main diagonal to the benign disease category. In this scenario, the agreement is increased for “Benign disease” category and mildly reduced for “Suspected cancer” category. The hypothetical cell counts under this scenario are represented with *†* symbol in Table 2.

Note that we impose no change on the remaining cells as the grey zone is only a concern for “Benign disease” and “Suspected cancer” categories for this data.

The resulting Cohen’s weighted Kappa values for the original data and the three hypothetical scenarios, given in Table 3, are computed using Eqs. (1), (2), and (3) and Table 4. Cohen’s weighted Kappa values are calculated using linear, quadratic and radical weights and an unweighted version of Kappa outlined in Table 5. In addition to different scenarios, the weighting scheme influences the value of agreement measures. Uniformly spaced linear or quadratic weights consider stronger disagreement for cells farther apart from the main diagonal. The quadratic weights return larger values than the linear weights; hence, they produce higher kappa values [19–21]. Warrens [19] gives the double inequality that “Cohen’s unweighted Kappa < Cohen’s linear weighted Kappa < Cohen’s quadratic weighted Kappa” which is also observed in Table 3. Cohen’s radical weighted Kappa sits in between unweighted and linear weighted version of Cohen’s Kappa in this example.

**Table 3:** The comparison across different scenarios of a grey zone in terms of Cohen’s weighted *κ* values for the motivating example

	Weight			
**Scenarios**	Unweighted	Linear	Quadratic	Radical
Original table	0.473	0.568	0.671	0.518
Scenario 1	0.206	0.343	0.504	0.270
Scenario 2	0.610	0.661	0.722	0.634
Scenario 3	0.551	0.612	0.685	0.579

**Table 4:**
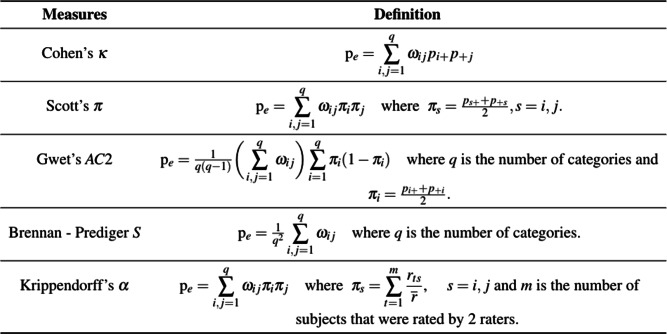
Definitions of *P*_*e*_ for the considered weighted measures

**Table 5:**
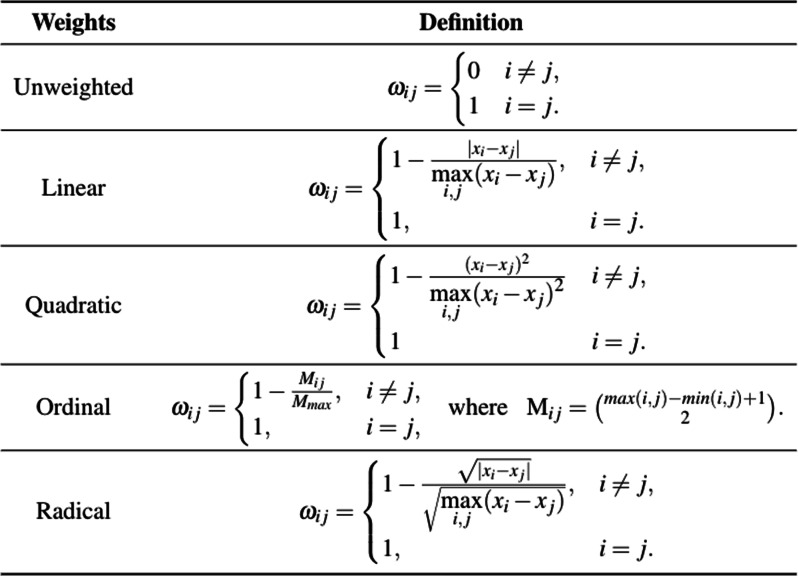
Weighting schemes and their definitions

The inter-rater agreement decreases in scenario 1 compared to the original table since the majority of Radiologist A’s suspected cancer ratings on the main diagonal are moved to the benign disease category. Such a movement caused inflation in the count of controversial class (i.e. Benign disease, suspected cancer). On the other hand, for scenario 2, the inter-rater agreement increases compared to the original table. This time, all of Radiologist A’s suspected cancer ratings which are not in the main diagonal are moved to the benign disease category. As expected, this movement increased the count of agreed class (i.e. Benign disease, Benign disease). Lastly, in scenario 3, we observe a decrease in agreement compared to scenario 2 and an increase as compared to the original table since some of the ratings on the main diagonal are moved to benign category. These hypothetical scenarios demonstrate how the degree of inter-rater agreement could change if Radiologist A rated less on the “Suspected cancer” category by fixing the marginals of Radiologist B. As seen, Radiologist A’s tendency to favour one of the diagnostic categories, which creates the grey zone, can alter the inter-rater agreement significantly. Therefore, studying and understanding the impact of grey zones on the inter-rater agreement measures is crucial to make reliable decisions.

## Methods

Suppose two independent raters code for all N subjects into a *q*×*q* contingency table. Let *m*_*ij*_ and *p*_*ij*_ signify the frequency distribution and the joint probability distribution, respectively, relating to *i*,*j*=1,2,…,*q* categories. Let *m*_*i*+_ and *m*_+*j*_ represent the marginal totals of the first and second rater. Also, the marginal probabilities are denoted by *p*_*i*+_ and *p*_+*j*_, reflecting the frequency use of the categories. Then, in the case of two raters, the inter-rater agreement of cross-classification for two raters is illustrated in Table 6.

**Table 6:** Classification of the inter-rater agreement between two raters

**Rater A**	Rater B				Total
	1	2	...	q	
1	*m*_11_	*m*_12_	...	*m*_1*q*_	*m*_1+_
2	*m*_21_	*m*_22_	...	*m*_2*q*_	*m*_2+_
...	...	...	...	...	...
q	*m*_*q*1_	*m*_*q*2_	...	*m*_*qq*_	*m*_*q*+_
Total	*m*_+1_	*m*_+2_	...	*m*_+*q*_	*N*=*m*_++_

### Inter-rater agreement measures

Let *δ* (−1≤*δ*≤1) represent the generic inter-rater agreement measure

1 $$ \delta = \frac{p_{a} - p_{e}}{1 - p_{e}},  $$

where *p*_*a*_ is the weighted proportion of observed agreement defined as

2 $$ p_{a} = \sum_{i=1}^{q}\sum_{j=1}^{q} \omega_{ij} p_{ij},  $$

and *p*_*e*_ is the weighted proportion of agreement expected by chance defined as

3 $$ p_{e} = \sum_{i,j=1}^{q} \omega_{ij} p_{i+} p_{+j}.  $$

The straightforward version of Cohen’s *κ* considers the classification variables as nominal and assumes that the effect of disagreement is uniform across all off-diagonal cells independently from their distance from the diagonal. When the classification is based on ordinal outcomes and/or the disagreement is stronger for larger distances between the ranking categories, the weighted version of Cohen’s *κ* is a measure that weights the disagreements according to their severity [22, 23]. Cohen’s *κ* [22, 24], Scott’s *π* [25], Gwet’s AC2 [26], Brennan - Prediger’s *S* [27], and Krippendorff’s *α* [28] are some of the common chance-corrected agreement measures to assess the agreement among two raters for ordinal data. For these measures, the main formula is as given in Eq. (1). However, Warrens [21] mentions that the definition of *p*_*e*_ differs for these measures as given in Table 4.

The main difference between the agreement measures in Table 4 is the definition of p _*e*_. Cohen’s weighted *κ* is a universal measure of agreement for ordinal outcomes, sensitive to the marginal distributions, and it does not correct disagreement [16, 29, 30]. Gwet’s AC2 can be used with all types of data and makes adjustments for the classification errors [29, 31]. Scott’s weighted *π* is applicable to ordinal outcomes and assumes that the raters have the same marginal distributions [30, 32]. All disagreements are treated equally by *κ* and *π*. However, Krippendorff’s *α* considers different magnitudes of disagreement and can be used with all types of data [30]. Brennan - Prediger’s *S* can be used with nominal, ordinal, ratio, and interval data and has the assumption that marginal distributions are uniform for the assessors rating by chance alone [29, 30].

Kappa statistics are interpreted in terms of the strength of agreement between the raters based on various classifications given in the literature. Different classifications in literature are tabulated by Yilmaz and Saracbasi [33, see Table 5 therein].

#### Weighting schemes

Suppose the *i*^*th*^ and *s*^*th*^ elements of row scores are denoted by *x*_*i*_ and *x*_*s*_ while the *j*^*th*^ and *t*^*th*^ elements of column scores are presented by *x*_*j*_ and *x*_*t*_ respectively, for $i, j, s, t = 1, 2, \dotsc, q$. Then, the definitions of the weighting schemes for ordinal agreement measures are as given in Table 5.

Linear weighting scheme assigns weights proportional to the disagreement between individual ratings while the quadratic weights are proportional to the square of the difference. In this way, quadratic weights assign higher values to disagreements than the linear weights and tend to increase as the table gets bigger. On the other hand, radical weights produce smaller weights for small disagreements than both the linear and quadratic weights. Since the ranks of ratings are taken into account by the ordinal weights, only their ranks are reflected in the weights. In terms of the interpretation of the value of kappa measures, the same classification should not be used for all the weighting schemes since some weights produce higher values than the others for the same agreement measure [19]. When the aim is to report a single agreement measure for ordinal outcomes, use of the linear weights instead of the quadratic weights is recommended by Warrens [19]. There are also some paradoxical situations where the quadratic weights fail to produce a reliable agreement measure [34]. Warrens [34] proves that i) when the number of categories is an odd number and marginal totals of the first and the last categories are equal for at least one of the raters, quadratic weighted *κ* is insensitive to the degree of agreement between the raters on the center category. ii) This situation extends to the middle row of the agreement table when there is a pattern where symmetric rows of the table are equal to each other and the number of categories is odd. The unweighted scheme in Table 5 does not take the magnitude of disagreements into consideration, whereas weighted agreement measures assign higher weights to the pairs of categories far from each other.

In general, the choice of weights in practice is related to the type of ratings. For the interval scale, the difference between two ratings is meaningful and there is an order among the ratings, whereas the order among the ratings is meaningful but the difference between them is not for the ordinal scale. The ratio scale has the features of the interval scale and there is a “true zero” in the scale (the variable is absent when it is equal to zero). Gwet [29, see Table 3.6.1] outlines the selection of a suitable weighting scheme based on the measurement scale. When the ratings are in the interval scale, the quadratic, linear, or radical weights are suitable. If the ratings are ordinal and they cannot be treated as interval or ratio data, only the ordinal weighting is a suitable choice. For ratio scale, the quadratic, linear, radical, ratio, circular, or bipolar weights are suitable [29]. In this study, we consider the weights given in Table 5.

### Table generation

The *q*×*q* contingency tables are generated by utilizing the underlying variable approach [35] that assumes the established ordinal variables are constructed by commonly distributed continuous latent variables. Algorithm 1 is used to generate a contingency table (with ordinal categories) given the correlation structure.

**Algorithm 1**

1. Input the total number of iterations.
2. Input the mean values for both variables, with *μ*_1_=0 and *μ*_2_=0; and the standard deviations *σ*_1_=*σ*_2_=1.
3. Input a pre-specified correlation structure *ρ*, for example, with a set 0.1,0.6,0.9 corresponding levels of the agreement such as low, medium, and high, respectively.
4. Randomly generate a pair of {*Y*_1*i*_,*Y*_2*i*_} bi-variate normal random variables with *i*=1,…,*n*. The values of means, standard deviations, and correlations are specified at the steps 1 to 3.
5. Input the number of categories, *q*. Use standard normal quantiles, *Φ*^−1^, as cut-offs for {*Y*_1*i*_,*Y*_2*i*_}. Three distinctive sets of structures are constructed to generate three different *q*×*q* contingency table structures, including balanced, slightly unbalanced and heavily unbalanced. To generate a *q*×*q* contingency table with balanced structure, the balanced set of $\Phi ^{-1} \{1/q, 2/q,\dots, (q-1)/q\}$ were taken as cut-offs for {*Y*_1*i*_,*Y*_2*i*_}. For the slightly and heavily unbalanced tables, see Tran et al. [16, p. 997].
6. Discretise {*Y*_1*i*_,*Y*_2*i*_} using standard normal distribution quantiles to ensure the ordinality of the generated variables and construct the joint probabilities (*p*_*ij*_) for all *q*×*q* tables.
7. Input the sample size *N*, for example from the set {*N*=50,100,200,500,1000}, and recall the R function “rTableICC.RxC” [36] with joint probability *p*_*ij*_ to generate table frequencies from multinomial distributions.

The true *κ* value is calculated by using the joint probabilities in *p*_*ij*_ for the calculation of goodness-of-fit measures for the assessment of the accuracy of agreement measures in a simulation setting.

### Creating grey zones

In this study, the existence of only one grey zone is taken into account. The single grey zone occurs when one of the raters tends to vote more on one of the categories. This is due to the fact that when both raters tend to vote more on the same category, the inter-rater agreement will increase and this will mask the potential impact of the grey zone.

To generate agreement tables having a grey zone, *p*_*ij*_ probabilities from step 6 of Algorithm 1 are used to generate a *q*×*q* contingency table with a grey zone by assuming that one of the raters tends to rate more on the subsequent category, namely the category *i* of *q*×*q* table that creates a grey category. So, they will move the count from other categories to the subsequent category *i* in terms of the cell counts in the table. For more than three categories, the movement cannot jump more than one category due to the observed ordinal variables are indicated by the corresponding normally distributed continuous variables as per Algorithm 1. To make category *i* a grey category, some frequencies from category (*i*−1) and category (*i*+1) are moved to category *i* by various rates. Let the rates of change from cell (*i*−1,*j*−1) to cell (*i*−1,*j*) and from cell (*i*+1,*j*+1) to cell (*i*+1,*j*) be *λ*_1_ and *λ*_2_, respectively. Then, the cell counts are calculated as in Eq. (4):

4 $$  \left\{\begin{array}{l} m_{(i-1)j} = m_{(i-1)j} + \lambda_{1} \cdot m_{(i-1)(j-1)}, \\ m_{(i+1)j} = m_{(i+1)j} + \lambda_{2} \cdot m_{(i+1)(j+1)}, \\ \end{array}\right.  $$

where *λ*_1_∈(0,1) and *λ*_2_∈(0,1). Then, we run a search over the space of (0,1)×(0,1) to find the particular values of *λ*_1_ and *λ*_2_ to make sure that the agreement in the generated table is equal to a given true *κ* value.

To be able to compare the results of simulations under the presence and absence of grey zones, the value of true agreement needs to be equivalent. Let *κ*_*I*_ be the true inter-rater agreement when there is no grey zones considered and *κ*_*II*_ be the true inter-rater agreement for the simulations under the existence of grey zones. Then, we need to make sure

5 $$ \kappa_{I} \approx \kappa_{II} \approx \kappa.  $$

We set the convergence criteria between the two simulations with and without a grey zone to satisfy Eq. (5) as in Eq. (6):

6 $$ \frac{1}{m} \sum_{i=1}^{m} |\kappa_{I(i)} - \kappa_{II(i)}| \leq \epsilon,  $$

where *ε* is small constant such as 0.01 and *m* is the total number of different kappa measures that are used to estimate the inter-rater agreement in the simulation spaces of both simulation I and simulation II.

Since the joint *p*_*ij*_ probabilities are used to find the realized value of true *κ*, at every iteration of the search, the counts are moved from cell to cell based on Eq. (4) until the condition in Eq. (6) is satisfied. At every iteration, we get a new set of joint probabilities $\widehat {p}_{ij}$. We propose two approaches for searching $\widehat {p}_{ij}$ with different starting probabilities. The first approach starts with joint probabilities corresponding to the perfect agreement called the PA approach, and the second approach uses a probability structure closest to the joint probability *p*_*ij*_ called the CP approach.

#### The PA approach

The PA or the perfect agreement approach uses each set of the cumulative probabilities for balanced and unbalanced structures mentioned in “Table generation” section to construct the main diagonal of the perfect agreement table with the same balanced or unbalanced structure. Table 7 describes the starting probability table having a perfect agreement.

**Table 7:** Probability structure for the perfect agreement of *q*×*q* tables with balanced structure

**Rater A**	Rater B				Total
	1	2	...	q	
1	1/*q*	0	...	0	1/*q*
2	0	1/*q*	...	0	1/*q*
...	...	...	...	...	...
q	0	0	...	1/*q*	1/*q*
Total	1/*q*	1/*q*	...	1/*q*	1

If we keep moving the cell counts from other categories to category *j* of rater B based on Eq. (5) without considering Eq. (6), the final probability structure turns out to be as given in Table 8. Since we migrate the cell counts from the neighbour cells to the category *j* of rater B, we create grey zones with different magnitudes, which create different true *κ* values, during the intermittent steps of the search.

**Table 8:** The final probability structure of *q*×*q* table for the last possible iteration of PA searching approach

	Rater B							
Rater A		1	2		s		q	Total
	1	0	0		1/q		0	1/q
	2	0	0		1/q		0	1/q
	q	0	0		1/q		0	1/q
	Total	0	0		1		0	1

In this approach, the non-zero counts are on the main diagonal of the table. Therefore, the cell counts just need to get moved from a cell on the main diagonal to a neighbour cell of the grey category. However, this search can be time-consuming for a low value of true *κ* because the initial values taken in this method imply the perfect agreement (*κ*=1). Another disadvantage of this approach is the existence of a lower limit for agreement value after completely moving all possible cells to grey category. For example, in a 6×6 contingency table with balanced structure, if the third category is identified as the grey category, the lower limit of agreement value is 0.6 after completely moving from cells (2,2) and (4,4) to cells (2,3) and (4,3), respectively. The initial table structures do not remain the same after searching. For instance, in a 3×3 table with balanced structure, the structure changes slightly to unbalanced after applying the PA method.

#### The CP approach

For the CP or the closest probability approach, we use a different cell count movement strategy than the one given in Eq. (4). We move a small probability *γ* from the diagonal cell, *m*_*ii*_ corresponding to the grey category *i*, to the next diagonal cell of the table in CP approach as outlined in Eq. (7).

7 $$ \left\{\begin{array}{l} m_{ii} = m_{ii} \mp \gamma \times m_{ii}, \\ m_{(i+1)(i+1)} = m_{(i+1)(i+1)} \pm \gamma \times m_{ii}, \\ \end{array}\right.  $$

where *γ*≈0.01 to keep the step size among the iterations of the search small to approach to a new closest joint probability structure, $p^{\prime }_{ij}$. Then, $p^{\prime }_{ij}$ is used for the search for the joint probabilities $\widehat {p}_{ij}$ that ensure the conditions in (5) and (6).

In the PA approach, the search is initiated from the perfect agreement while the search starts using the cell counts of the given table in the CP approach. Therefore, this approach requires a less number of iterations to reach out a low degree of agreement in the search; hence, it is computationally more efficient than the PA approach to generate a low or moderate true degree of agreement. In this approach, there is an increase in the number of possible cells to move to the grey category because all counts can be non-zero. For example, in *q*×*q* tables, if rater B has a grey zone in category *i*, not only the counts from the cell (*i*−1,*i*−1) but also those from the cell (*i*−1,*i*+1) can be moved into the cell (*i*−1,*i*). Similarly, both cells (*i*+1,*i*+1) and (*i*+1,*i*−1) can transfer counts to cell (*i*+1,*i*) in this situation.

In conclusion, the CP approach is used to search for all $\widehat {p}_{ij}$ values. Then, *p*_*ij*_ and $\widehat {p}_{ij}$ are used to generate two sets of *q*×*q* contingency tables by taking Algorithm 1 in “Table generation” section. For each scenario, the data table does not have a grey zone if it is generated by using *p*_*ij*_, has a grey zone if it is generated by using $\widehat {p}_{ij}$.

## Results

This section describes a comprehensive Monte Carlo simulation study throughout various methods of weighted inter-rater agreement measures and weights with regards to two raters for ordinal outcomes. The simulation is run for both cases of having a grey zone and no grey zone.

### Simulation space and data generation

The simulation space of our Monte Carlo study is composed of 4500 combinations of three table structures, five different sample sizes, four various numbers of categories and three levels of actual inter-rater agreements. The considered sample sizes include small (*n*=50), moderate (*n*=100 and 200), large (*n*=500), and very large (*n*=1000) agreement tables. Small to moderately sized agreement tables are common in the health and education fields. Large and very large agreement tables are also of interest in health information management [37]. We implement five different weights with each of five inter-rater agreement measures outlined in Table 9. By this extensive simulation space, a distinct evaluation of the impact of having a grey zone on the weighted inter-rater agreement measures is presented.

**Table 9:** Levels and abbreviations of the factors constituting the simulation space, inter-rater agreement measures and weights

*R*×*R* table	Abbr.	Level of true agreement	Abbr.	Table structure	Abbr.
*q*=3	3×3	Low	L	Balanced	BL
*q*=4	4×4	Medium	M	Slightly Unbalanced	UB1
*q*=5	5×5	High	H	Heavily Unbalanced	UB2
*q*=6	6×6				
**Sample size**	**Abbr.**	**Inter-rater agreement measure**	**Abbr.**	**Type of weight**	**Abbr.**
50	*n*=50	Cohen’s kappa	*κ*	Unweighted	unweighted
100	*n*=100	Scott’s *π*	*π*	Linear weight	linear
200	*n*=200	Gwet’s AC2	AC2	Quadratic weight	quadratic
500	*n*=500	Brennan - Prediger	BP	Ordinal weight	ordinal
1000	n=1000	Krippendorff’s *α*	*α*	Radical weight	radical

As described in Algorithm 1, underlying latent variables for the generation of ordinal agreement tables are created by using the bivariate normal distribution and the Pearson correlation coefficient (*ρ*) to set the values of true inter-rater agreement among observers. The true agreement (not chance-corrected) is adequately quantified by using the latent variables and the predetermined correlation structure when both variables were examined on the same rate. At step 3 of Algorithm 1, *ρ* is set to 0.1,0.6, and 0.9 to create low, medium, and high degrees of agreement, respectively. The balanced, slightly unbalanced, and heavily unbalanced table structures are created at step 5 of Algorithm 1. Then, the joint probability structure is constructed at step 6 and used to generate the cell counts of the randomly generated agreement table at step 7 of Algorithm 1. The actual (population) inter-rater agreement coefficients were calculated using *p*_*ij*_’s constructed at step 6 of Algorithm 1. The actual inter-rater agreement coefficient is denoted as *κ*.

The joint probability $\widehat {p}_{ij}$ and *p*_*ij*_ were taken to generate all *q*×*q* contingency tables, without (simulation I) and with (simulation II) a grey zone respectively, across scenarios mentioned in the simulation design given in Table 9. Then, the first simulation was run by using the tables that do not have a grey zone before doing the second simulation taking the tables with a grey zone.

To compare the results between simulation I and simulation II, the mean absolute error (MAE), the mean square error (MSE), and the mean absolute percentage error (MAPE) are used as goodness-of-fit measures. For the search methods of “Creating grey zones” section, both condition (5) and (6) are required to be satisfied. Therefore, the true inter-rater agreement coefficients *κ* is approximately the same for both simulation I and II. The MAE values for the first simulation, *Δ*_1_, and the second simulation, *Δ*_2_, are estimated using Eqs. (8) and (9) respectively as follows:

8 $$  \Delta_{1} = \frac{1}{r} \sum_{i=1}^{r} |\kappa - \hat{\kappa}^{\prime}_{i}|,  $$

9 $$  \Delta_{2} = \frac{1}{r} \sum_{i=1}^{r} |\kappa - \hat{\kappa}^{\prime\prime}_{i}|,  $$

where, *i*=1,...,*r* is the number of replications and *κ* is the true inter-rater agreement coefficient. $\hat {\kappa }^{\prime }_{i}$ and $\hat {\kappa }^{\prime \prime }_{i}$ are the inter-rater agreement estimation in the *i*th replication of the first simulation and the second simulation, respectively.

### Simulation results

**Fig. 1:**
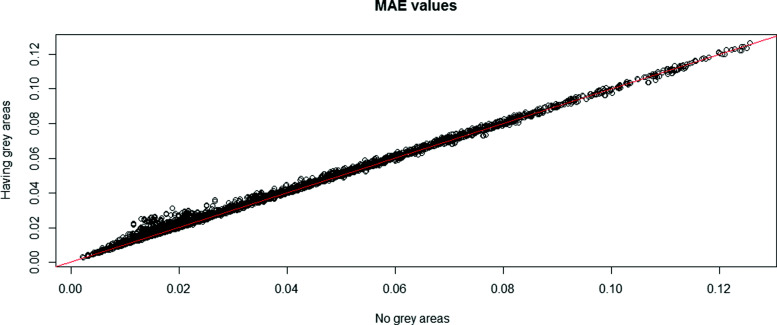
The overall comparison of MAE values from two simulations with and without the existence of a grey zone. The dots show MAE values averaged over all simulation scenarios. Having dots in the upper side of the 45-degree line implies that the existence of a grey zone in the table increases MAE values

**Fig. 2:**
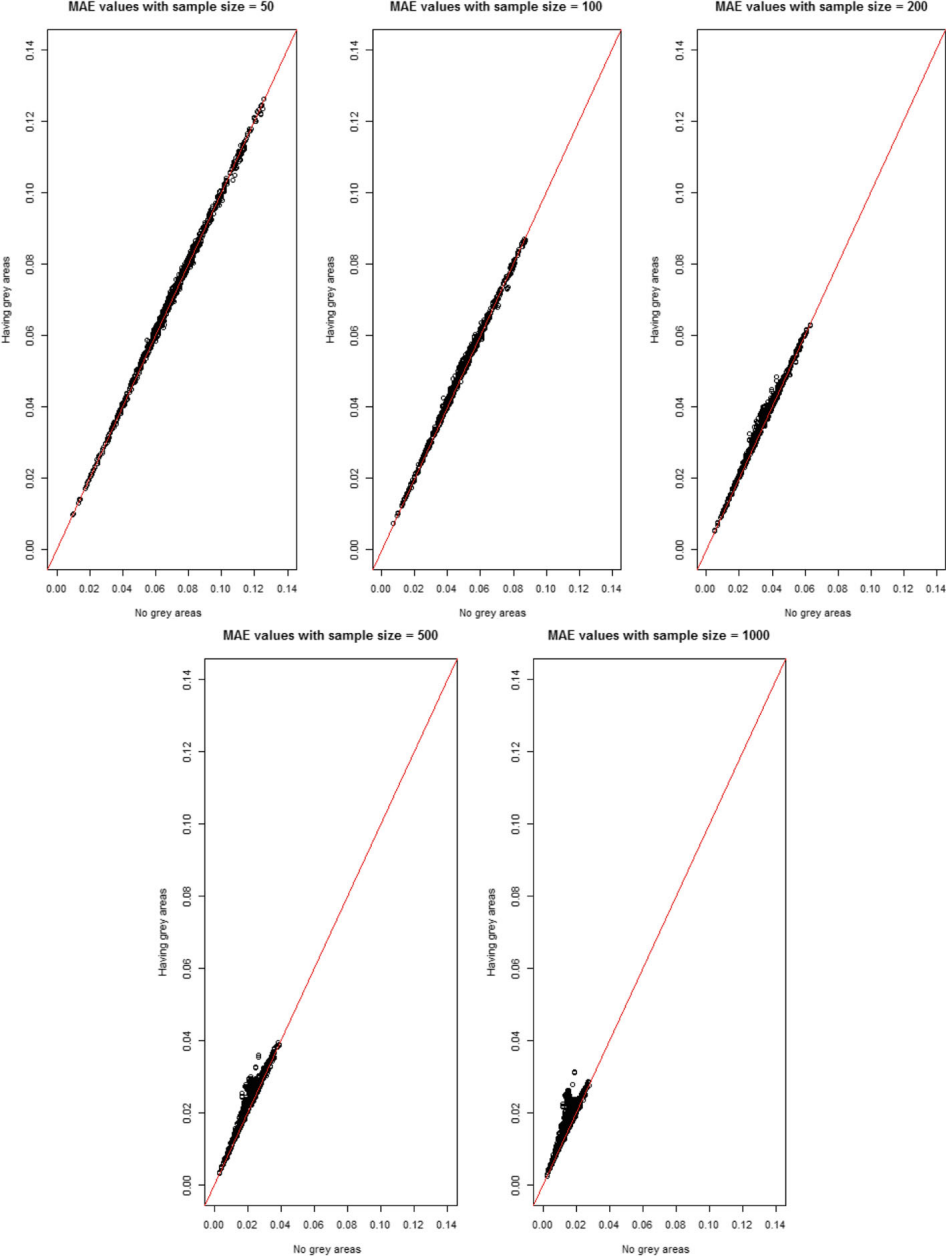
The comparison of MAE values from two simulations with and without the existence of a grey zone for the sample size of 50, 100, 200, 500, and 1000. Having more dots in the upper side of the 45-degree line for higher sample sizes implies that tables with larger samples are more impacted by the existence of a grey zone than the smaller samples

**Fig. 3:**
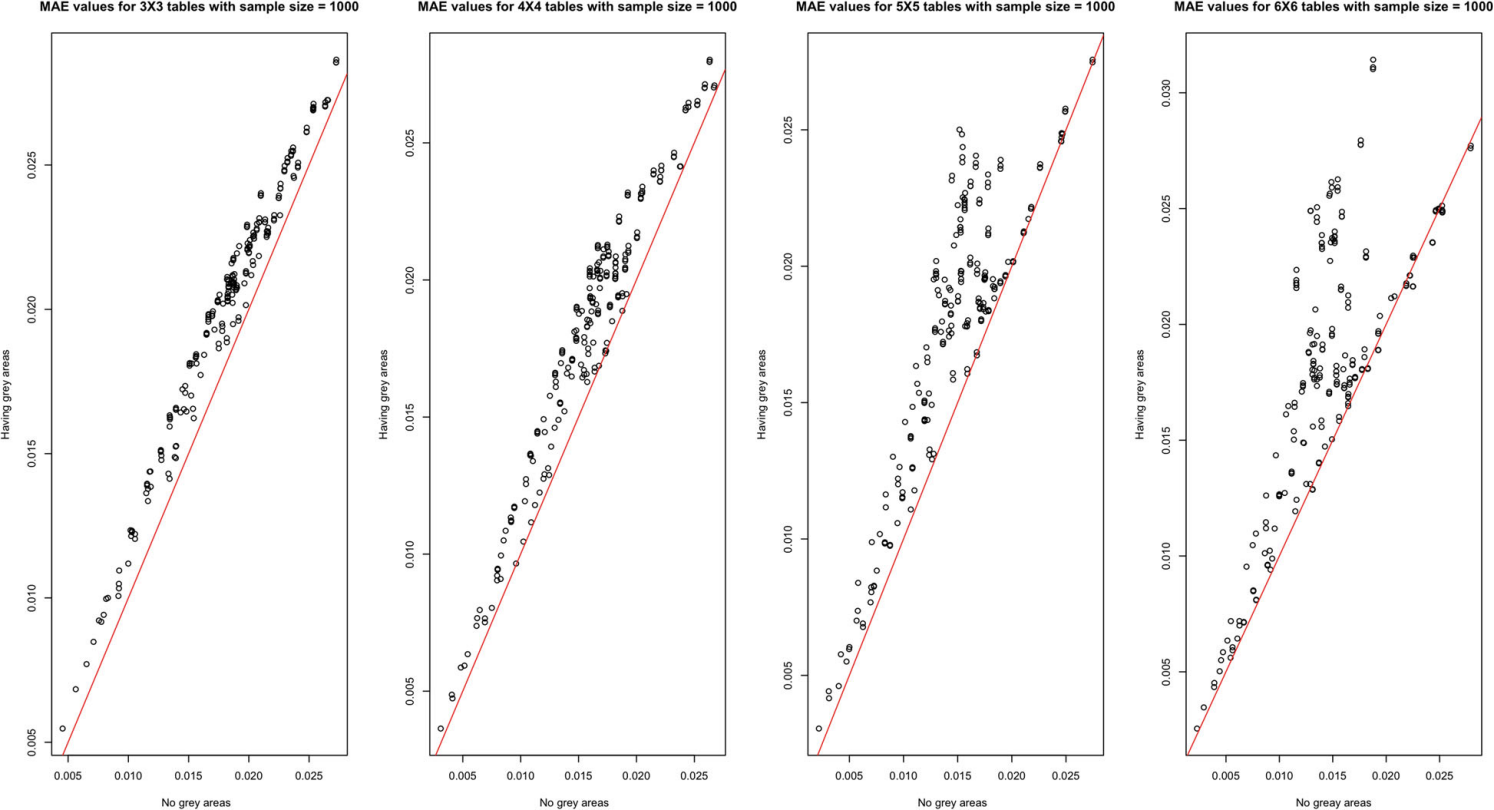
The comparison of MAE values from two simulations with and without the existence of a grey zone for the sample size of 1000 and 3x3, 4x4, 5x5, and 6x6 table sizes. For all table sizes, most of the dots are in the upper side of the 45-degrees line showing the impact of having a grey zone in the table to worsen the MAE values

**Fig. 4:**
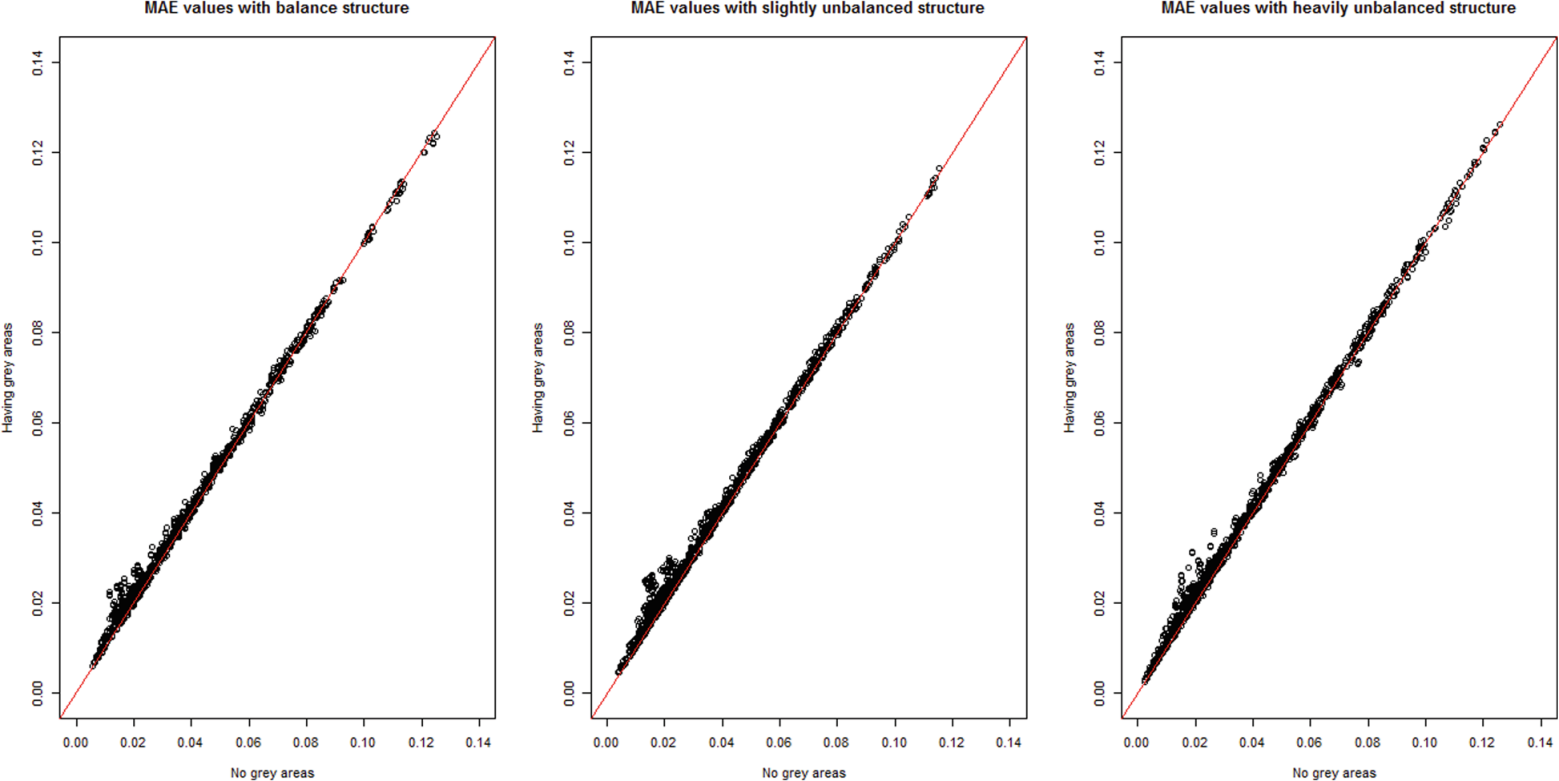
The comparison of MAE values from two simulations with and without the existence of a grey zone for balanced, slightly unbalanced, and highly unbalanced table structures. For all three cases of table structure, dots in the upper side of the 45-degree line imply that the existence of a grey zone in the table increases MAE values

**Fig. 5:**
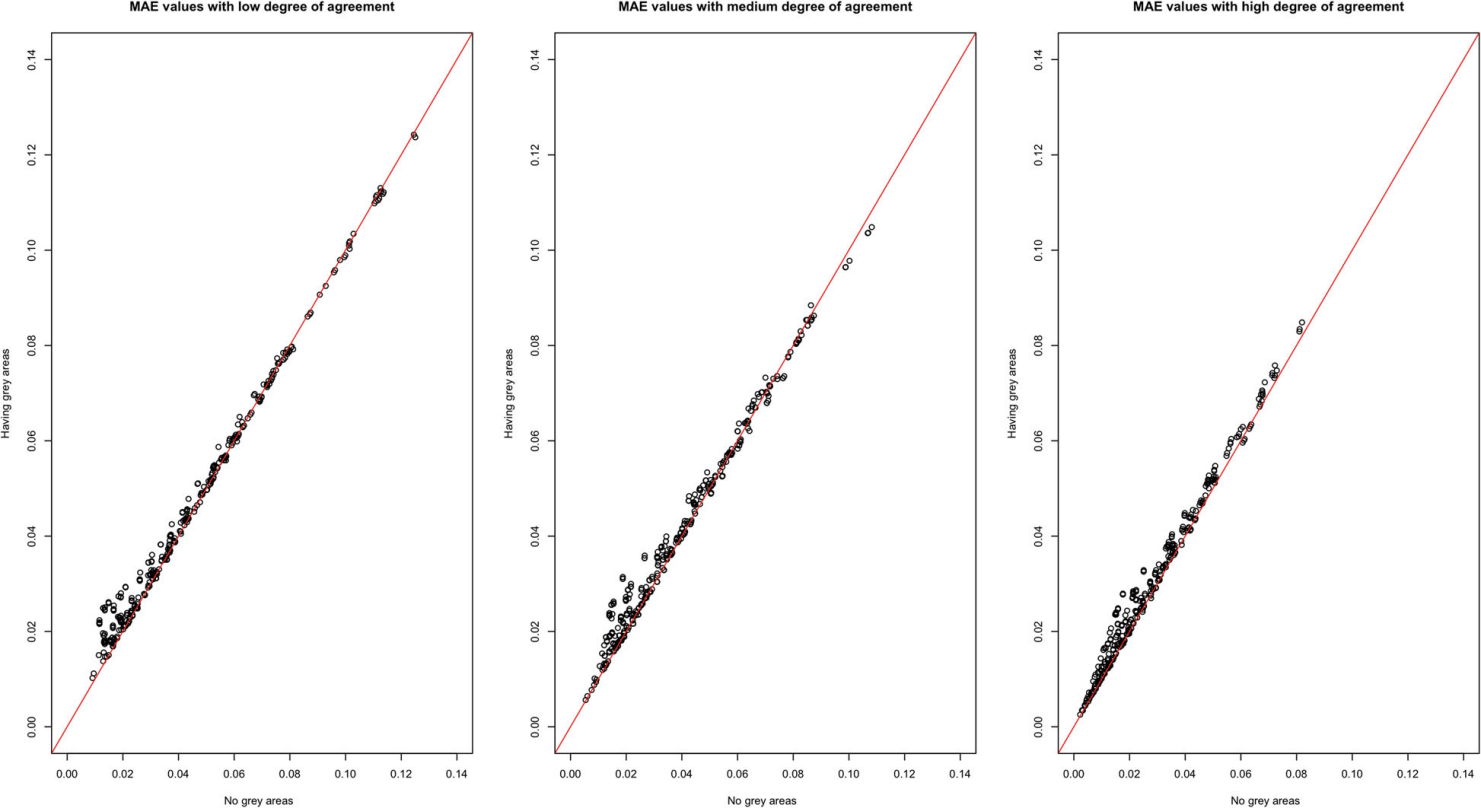
The comparison of MAE values from two simulations with and without the existence of a grey zone for low, medium, and high degree of true agreement. The considerable number of dots that are in the upper side of the 45-degrees line shows the impact of having a grey zone in the table to increase the MAE values

Because of the interpretations of the results over MAE, MSE, and MAPE are similar, only the MAE results are shown in this section across all scenarios. In Figs. 1, 2, 3, 4 and 5, the comparison of the MAE values of simulation I to those of simulation II are presented. In these figures, the plotted red line shows the 45-degrees line. When the points are above the red line, this implies that MAE is increasing when we have grey zones in the table. In this section, we use the term “accuracy” to refer to the desired cases of MAE, MAPE, and MSE. For example, a high accuracy corresponds to low values of these statistics.

Form Figs. 1, 2, 3, 4 and 5, we can conclude in general that having a grey zone in one category of an agreement table produces less accurate results. In these figures, most of the values are in the upper side of the red line, implying that MAE values of data having a grey zone are greater than those of data without a grey zone in the general sense. Specifically, since the number of points in the upper side of the 45-degrees line increases, the accuracy of the agreement measures decreases with increasing sample sizes (Fig. 2), the number of categories (Fig. 3 and 9 to 12 of Supplementary Material), and the degree of true agreement (Fig. 5) when these factors are considered solely and independent of each other. The unbalancedness in the cell counts of all considered ordinal tables negatively impacts the accuracy of inter-rater agreement measures (Fig. 4).

From Fig. 2, we observe that the degree of the negative impact of having a grey zone increases when the sample gets larger while it is not quite impactful with a sample of size 50. This is an expected result since the amount of information on the grey zone in a small sample is not high enough to be observed.

Figure 3 and 9 to 12 of Supplementary Material show the whole picture of MAE values in combinations of sample sizes and the number of categories. The impact of a grey zone on the accuracy of the agreement measures rises with the increasing sample size and number of categories. When the sample size is 50, the negative impact of having a grey zone gets higher as the number of categories increases. However, for the sample sizes greater than 50, most of the points are above the red line for all of the table sizes implying a significant negative impact of having a grey zone. For the sample sizes of 500 and 1000, even the agreement measures computed over 3x3 tables are being impacted by the grey zone.

Figure 4 shows the comparison between the two simulations in terms of the table structure. For all the table structures, we observe a similar pattern where higher MAE values are seen when a grey zone is introduced into the table for a considerable proportion of the simulation runs.

Figure 5 and 13 to 15 of Supplementary Material show the impact of having grey zones for the degree of true agreement and the table size. In general, having grey zones has a similar impact across low, moderate, and high degrees of agreement. The magnitude of their impact increases along with increasing table size.

**Fig. 6:**
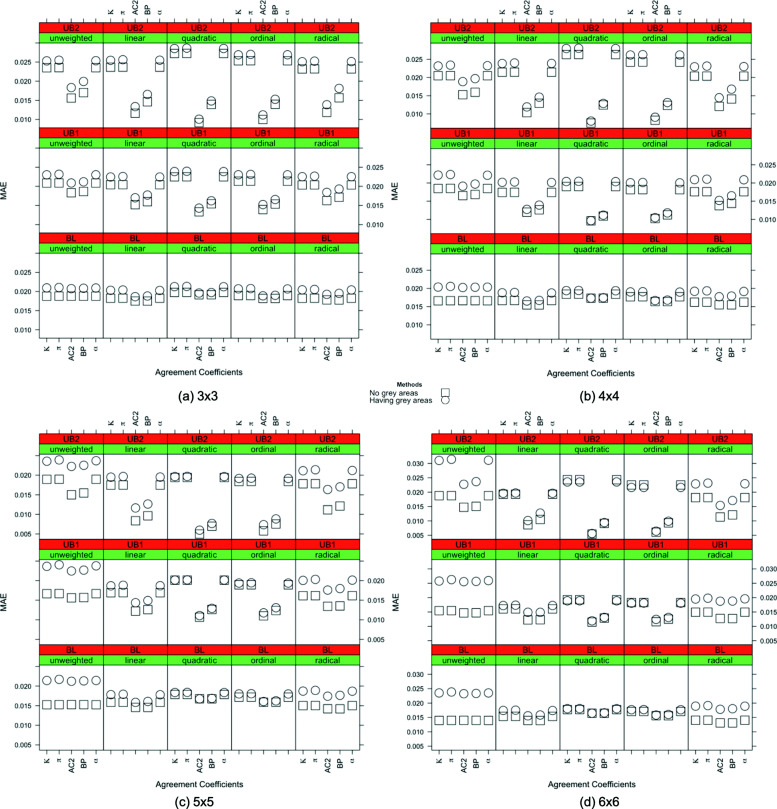
*Low degree of true agreement*: the MAE values for inter-rater agreement coefficients with sample size n = 1000 are given for all combinations of the weights, the degree of unbalancedness and the table size with and without the existence of a grey zone in the table when the degree of true agreement is low

**Fig. 7:**
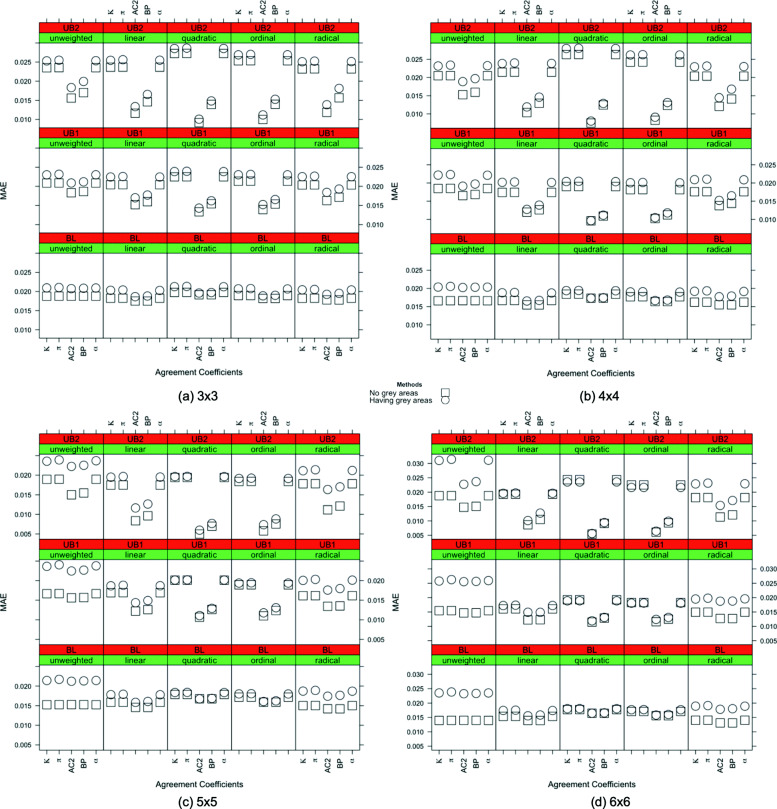
*Medium degree of true agreement*: the MAE values for inter-rater agreement coefficients with sample size n = 1000 are given for all combinations of the weights, the degree of unbalancedness and the table size with and without the existence of a grey zone in the table when the degree of true agreement is medium

**Fig. 8:**
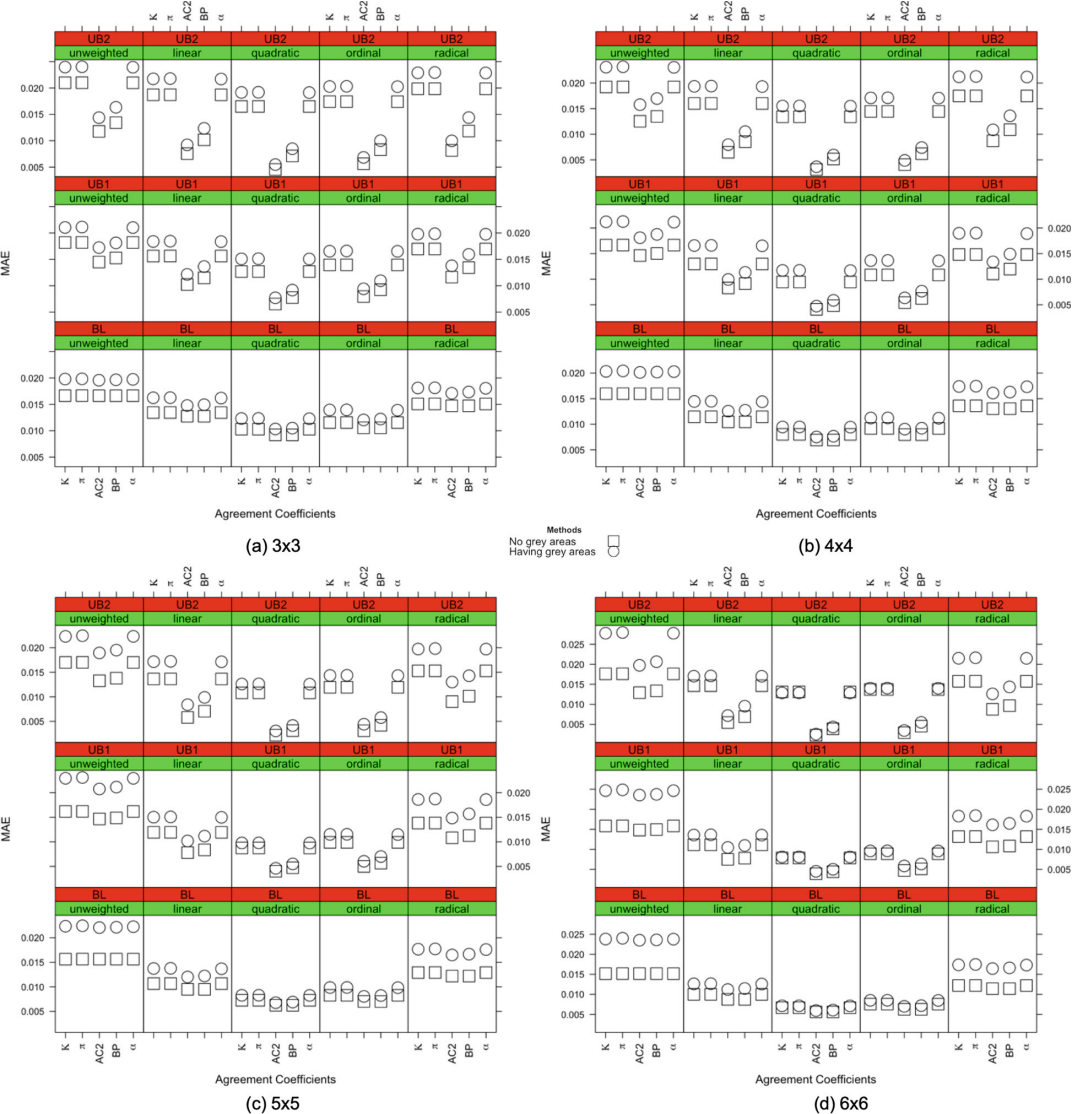
*High degree of true agreement*: the MAE values for inter-rater agreement coefficients with sample size n = 1000 are given for all combinations of the degree of unbalancedness and the table size with and without the existence of a grey zone in the table when the degree of true agreement is high

For low, medium, and high degrees of agreement, the MAEs of individual inter-rater agreement measures are given in Figs. 6, 7 and 8. Compared to the quadratic weighted *κ*,*π* and *α* unweighted version of the measures, which is not considered to be a proper choice for ordinal variables, produce more accurate results only for low agreement cases in 3×3 and 4×4 tables. On the other hand, for the high degree of agreement, unweighted measures produce less accurate results than the others while quadratic weights produce more accurate outcomes.

Moving on to the inter-rater agreement measures, the MAE values for all the measures are displayed for all combinations of the weights, the degree of unbalancedness, the table size, and the degree of true agreement in simulation I and simulation II in Figs. 6 to 8 for the sample size of 1000. For smaller samples, we observe similar patterns except *n*=50 where we do not see a significant impact of grey zones. In general, AC2 and BP measures produce close results to the no-grey-zone case under the existence of a grey zone in most of the cases, except the low degree of agreement with balanced structures. In these cases, AC2 and BP measures make less accurate outcomes when using quadratic and ordinal weights. In Fig. 6, when the strength of the true agreement is low, for 3×3 tables, the accuracy of the coefficients are close to each other in both simulations for the unweighted and linear, quadratic, and ordinal weighted measures under the balanced and unbalanced table structures. Those with radical weights perform worst under the existence of grey zones. When the size of the table gets bigger, unweighted and radical weighted measures perform worse while linear, quadratic and ordinal weights produce more accurate results. For 5×5 and 6×6 tables, the linear, quadratic, and ordinal weighted measures are not impacted by the existence of grey zones for balanced and unbalanced tables when the degree of true agreement is low. The use of AC2 and BP coefficients gives the most accurate MAE results across the considered combinations of unbalancedness, weights and table sizes when the degree of true agreement is low.

When the degree of true agreement is medium, the MAE values under the absence and presence of a grey zone are given in Fig. 7. The patterns seen in Fig. 7 are similar to those of Fig. 6 for all the weights. For unweighted tables, the impact of having a grey zone is slightly less for larger tables when the degree of true agreement is increased to medium. When the table size is increased, the linear, quadratic, and ordinal weights give more accurate results for all unbalancedness scenarios. When the table size is smaller, the unbalancedness of the table has an impact on the accuracy of these weights. Again the smallest MAEs are obtained for AC2 and BP measures for medium degree of true agreement in general.

Figure 8 shows the MAE values of the inter-rater agreement measures obtained for the high degree of true agreement. The difference between the MAE results of two simulations gets lower when we have a high degree of agreement. This is an expected result validating the suitability of simulations in the sense that when there is a high degree of agreement between raters, the grey zone becomes negligible and the accuracy of the simulations becomes closer.

## Discussion

When there is no gold standard diagnosis available, the reliability of diagnosis is often of interest. It is assessed by the degree of inter-rater agreement in a contingency table where a grey zone would occur when the raters tend to rate more in one category than another. As shown in “Motivating example: diagnostic classification of films for breast cancer” section, a rater can place more ratings in one of the categories in an attempt to make a safer diagnosis or avoid missing out severe cases. Such behaviour can be related to following different guidelines or lack of uniform protocols for rating [6, 7] as well as the subjective characteristics such as diagnostic styles of raters, habits shaped/learned in the clinic or even the rater’s elaboration of the diagnostic criteria [38]. Our simulation results show that the accuracy of agreement among raters decreases when the grey zone exists. Therefore, the existence of a grey zone can lead to a wrong decision about the reliability of diagnosis or instruments etc. based on the strength of inter-rater agreement; and hence, it should be taken into consideration as an important factor affecting the inter-rater agreement.

In this paper, we provide a definition of a grey zone in the inter-rater agreement context. There are studies in the literature suggesting that the marginal distributions of raters have an impact on the agreement [27, 39–41], but these studies do not associate “the difference in marginal distributions” to a “grey zone effect” as done in this study. Moreover, we propose two iterative methods to generate ordinal tables with a grey zone specified by the rate of migration between adjacent categories. We base our simulation study on these methods and by using these methods, we assess the effect of different type of grey zones, table structures, true agreement levels, and the sample sizes on the accuracy of the weighted inter-rater agreement measures for ordinal tables.

We also observe that the effect of grey zones on the accuracy of inter-rater agreement measures are associated with the degree of the true agreement. Similarly compared to the results in Tran et al. [16], all agreement measures with balanced structures produce more accurate outcomes than those with unbalanced structures and it is suggested to use Gwet’s AC2 and Brennan-Prediger’s *S* in combination with quadratic and ordinal weights to be on the safe side under the possibility of having a grey zone in the agreement table. A similar observation was made by Quarfoot and Levine [42] in inter-rater reliability context. Brennan-Prediger’s *S* assumes that the marginal distributions of raters are uniform [30]; and hence, it does not include the marginal probabilities in its formulation. Gwet’s *AC*2 only includes marginal probabilities of each category. Both *S* and *AC*2 rely on neither homogeneity of marginals as in Scott’s *π* nor heterogeneity of marginals as in Krippendorff’s *α*. These features of *AC*2 and *S* measures make them more reliable against the variations in the marginal distributions due to the existence of grey zones.

To clarify and further discuss the main findings, we revisit the motivating example of “Motivating example: diagnostic classification of films for breast cancer” section and calculate the weighted agreement measures with the data in Table 1 and hypothetical scenarios in Table 2. Kappa values and coefficient of variation (CV, standard deviation divided by mean) for each weighted agreement measure are reported in Table 10. CV is calculated over the Kappa values for the original table and three scenarios to see the magnitude of change across different realisations of grey zone in the agreement table. The smallest CV is obtained for quadratic weighted Gwet’s *AC*2. Gwet’s *AC*2 produced the smallest CV under each weight and *AC*2 is followed by Brennan-Prediger’s *S*. This observation with the motivating example data is consistent with the results of our simulation study and theoretical characteristics of *AC*2 and *S*.

**Table 10:** Weighted inter-rater agreement measures for the motivating example

		Weight				
Measure	Table	Unweighted	Linear	Quadratic	Radical	Ordinal
Scott’s *π*	Original	0.461	0.564	0.671	0.510	0.629
	Scenario 1	0.258	0.367	0.503	0.308	0.447
	Scenario 2	0.504	0.582	0.671	0.541	0.635
	Scenario 3	0.469	0.552	0.648	0.508	0.610
	CV	0.263	0.194	0.130	0.229	0.154
Gwet’s *AC*2	Original	0.529	0.719	0.850	0.629	0.809
	Scenario 1	0.407	0.673	0.840	0.550	0.790
	Scenario 2	0.576	0.750	0.869	0.668	0.832
	Scenario 3	0.550	0.737	0.863	0.649	0.825
	CV	0.145	0.047	0.015	0.083	0.023
Krippendorff’s *α*	Original	0.464	0.566	0.673	0.513	0.631
	Scenario 1	0.262	0.370	0.506	0.312	0.450
	Scenario 2	0.507	0.584	0.673	0.543	0.637
	Scenario 3	0.472	0.554	0.650	0.510	0.612
	CV	0.260	0.192	0.129	0.226	0.153
Brennan-Prediger’s *S*	Original	0.514	0.680	0.812	0.599	0.768
	Scenario 1	0.376	0.600	0.774	0.491	0.716
	Scenario 2	0.560	0.711	0.830	0.637	0.790
	Scenario 3	0.532	0.694	0.821	0.615	0.779
	CV	0.165	0.073	0.030	0.111	0.043

CV: coefficient of variation

Coefficient of variation is calculated for each pair of agreement measure and weight

## Conclusions

The accuracy of kappa coefficients in combination with 5 weighting schemes across scenarios for ordinal tables, as described in Table 9, is evaluated to show the effects of grey zones on the accuracy of inter-rater agreement measures. Main findings of our simulation study are summarized as follows:

- When a grey zone exists, the accuracy of inter-rater agreement measures reduces if the strength of true agreement between the raters is not high.
- The effect of grey zones on the accuracy of inter-rater agreement measures is associated with the degree of true agreement. When the degree of agreement increases, the accuracy of inter-rater agreement measures increases, but the impact of a grey zone on the accuracy of the inter-rater agreement measures decreases.
- Under the high degree of true agreement, when the table structure is balanced, all the measures produce similar results with quadratic and ordinal weights and most accurate ones are AC2 and *S* measures. Therefore, we recommend use of Gwet’s AC2 or Brennan-Prediger’s *S* with quadratic weights for interval ratings and ordinal weights for purely ordinal ratings when the agreement table is balanced and the main diagonal of the table has a considerable amount of frequencies implying a high degree of agreement.
- Increasing table size strengthens the information on the degree of agreement; and hence, reduces the discrepancy between the cases of no-grey-zone and having grey zones in ordinal tables.
- The quadratic and ordinal weights produce more accurate results with Gwet’s AC2 and Brennan-Prediger’s *S* measures in most of the simulations having and not having a grey zone. Thus, the use of AC2 or *S* measures with quadratic and ordinal weights, respectively for interval and ordinal ratings, is recommended if there is a possibility of having grey zones.
- The accuracy of inter-rater agreement measures increases with the increasing number of categories but decreases with increasing sample size when there is a grey zone in the table.
- All agreement coefficients with balanced structures produce more accurate outcomes than those with unbalanced structures under the absence and presence of grey zones.
- Gwet’s AC2 and Brennan-Prediger’s *S* in combination with quadratic or ordinal weights produce more accurate results except for the low degree of agreement for balanced structure.

After the estimation of a global agreement measure, the next step is the partial agreement between levels of the ordinal outcome. In this study, we demonstrated the sensitivity of ordinal agreement to variations in the agreement among consecutive ordinal levels. As a future study, it would be valuable to develop an approach that utilises the variation among ordinal levels as an indication of the existence of grey zones.

## Appendices

## Additional simulation results

Additional simulation results, presented in Figs. 9 to 15, are given in the Supplementary Material that can be found online at 10.1186/s12874-021-01248-3.

## Supplementary Information

**Additional file 1** Supplementary Material for The impact of grey zones on the accuracy of agreement measures for ordinal tables.

## Data Availability

There is no administrative permissions are required to access the data used in this study. The datasets used and/or analysed during the current study are available from the first author or corresponding author on request. Declarations

## Abbreviations

PA: Perfect agreement
CP: Closest probability
MAE: Mean absolute error
MSE: Mean squared error
MAPE: Mean absolute percentage error
CV: Coefficient of variation
L: Low agreement
M: Medium agreement
H: High agreement
BL: Balanced
UB1: Slightly unbalanced
UB2: Highly unbalanced
*κ*: Cohen’s kappa
*π*: Scott’s *π*
AC2: Gwet’s AC2
BP: Brennan - Prediger
*α*: Krippendorff’s *α*
Unweighted: Unweighted
Linear: Linear weight
Quadratic: Quadratic weight
Ordinal: Ordinal weight
Radical: Radical weight.
*γ*: A small probability
