# Supplementary material for: The impact of grey zones on the accuracy of agreement measures for ordinal tables
Source: BMC Med Res Methodol. 2021 Apr 14;21:70. doi: 10.1186/s12874-021-01248-3 (PMC8048180; doi:10.1186/s12874-021-01248-3)
Supplement: Supplementary file 1 — Additional file 1 Supplementary Material for The impact of grey zones on the accuracy of agreement measures for ordinal tables. [file 12874_2021_1248_MOESM1_ESM.pdf]

Supplementary Material for The impact of grey zones on the accuracy of agreement measures for ordinal tables

Quoc Duyet Tran
An Giang University, VNU-HCM, Long Xuyen City, An Giang Province, 076, Vietnam
Mathematical Sciences, School of Science, RMIT University, Melbourne, Victoria, 3000, Australia
duyet.tran@rmit.edu.au

Anil Dolgun
Mathematical Sciences, School of Science, RMIT University, Melbourne, Victoria, 3000, Australia
anildolgun@gmail.com

Haydar Demirhan
(Corresponding author)
Mathematical Sciences, School of Science, RMIT University, Melbourne, Victoria, 3000, Australia
haydar.demirhan@rmit.edu.au

1 Additional simulation results

The MAE values for the two simulations are plotted against each other for table sizes of 3×3, 4×4, 5×5, and 6×6 and sample sizes of 50, 100, 200, and 500 in Tables 9 to 12, respectively.

The comparison of MAE values of two simulations over low, medium, and high degrees of agreement is given in Figure 13 and those are given in the breakdown of low and medium degrees of agreement and the table sizes in Figure 14 and 15, respectively.

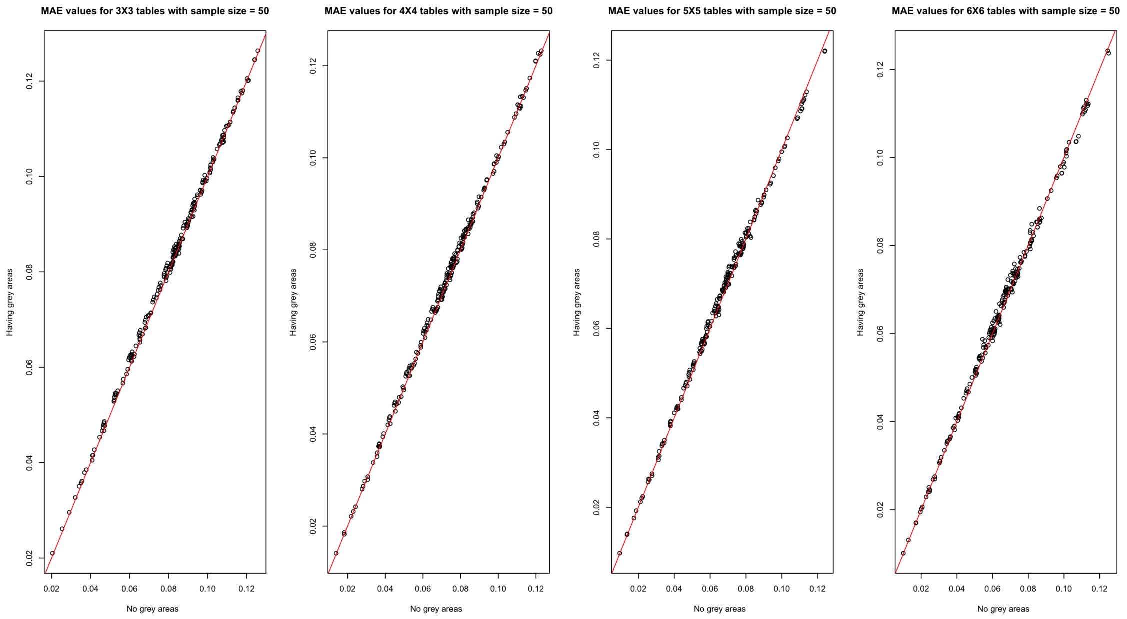

Figure 9: The comparison between two simulations for the sample size of 50 with 3x3, 4x4, 5x5, and 6x6 table sizes.

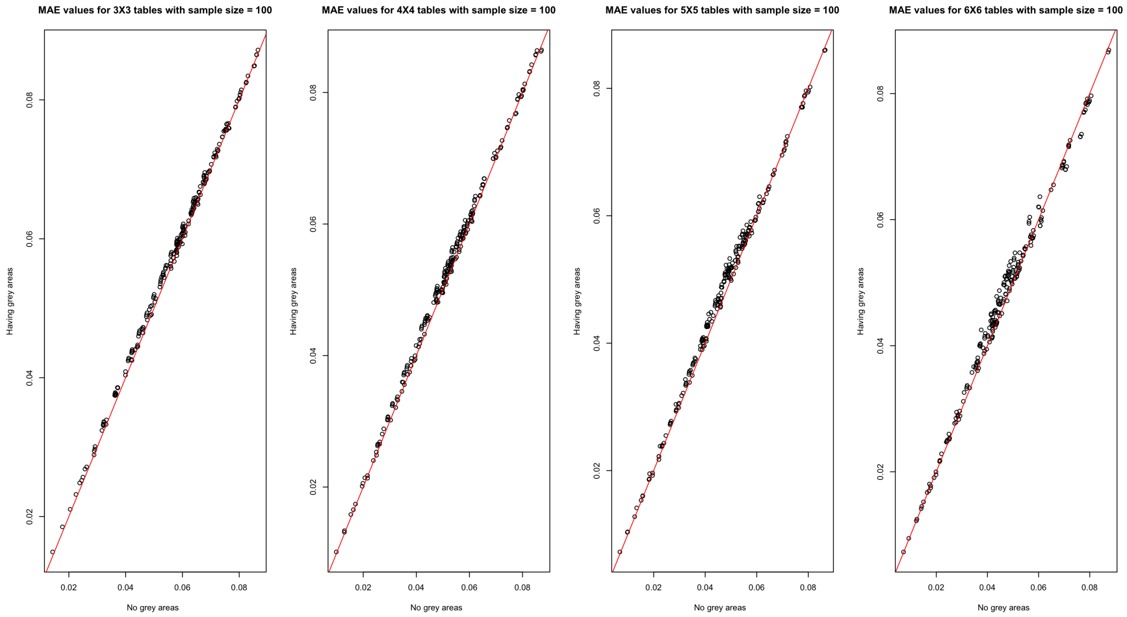

Figure 10: The comparison between two simulations for the sample size of 100 with 3x3, 4x4, 5x5, and 6x6 table sizes.

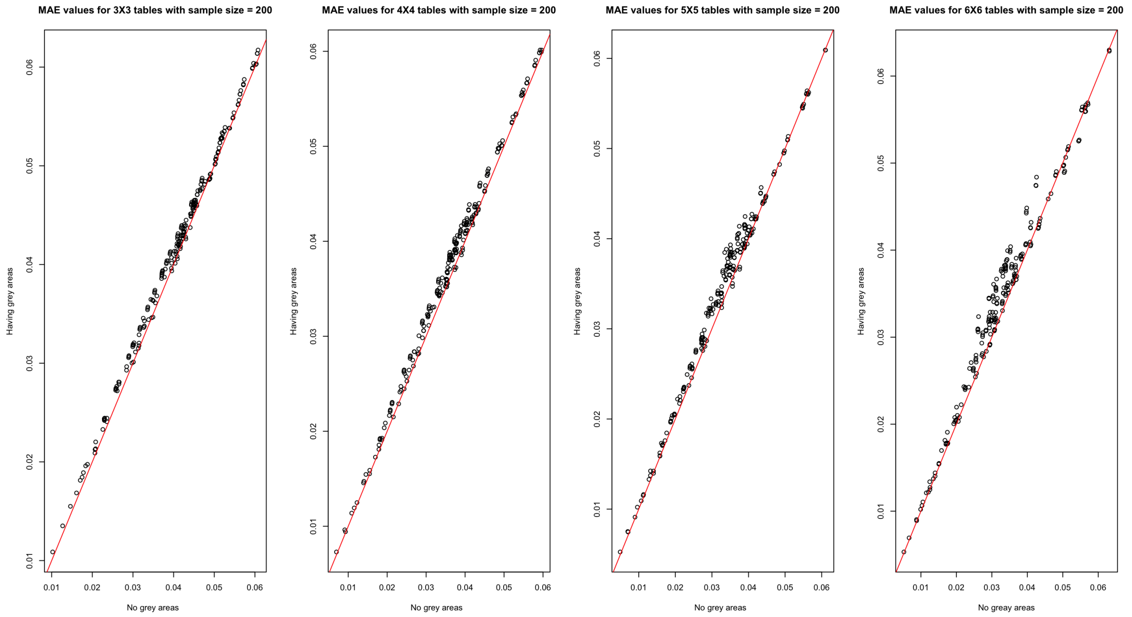

Figure 11: The comparison between two simulations for the sample size of 200 with 3x3, 4x4, 5x5, and 6x6 table sizes.

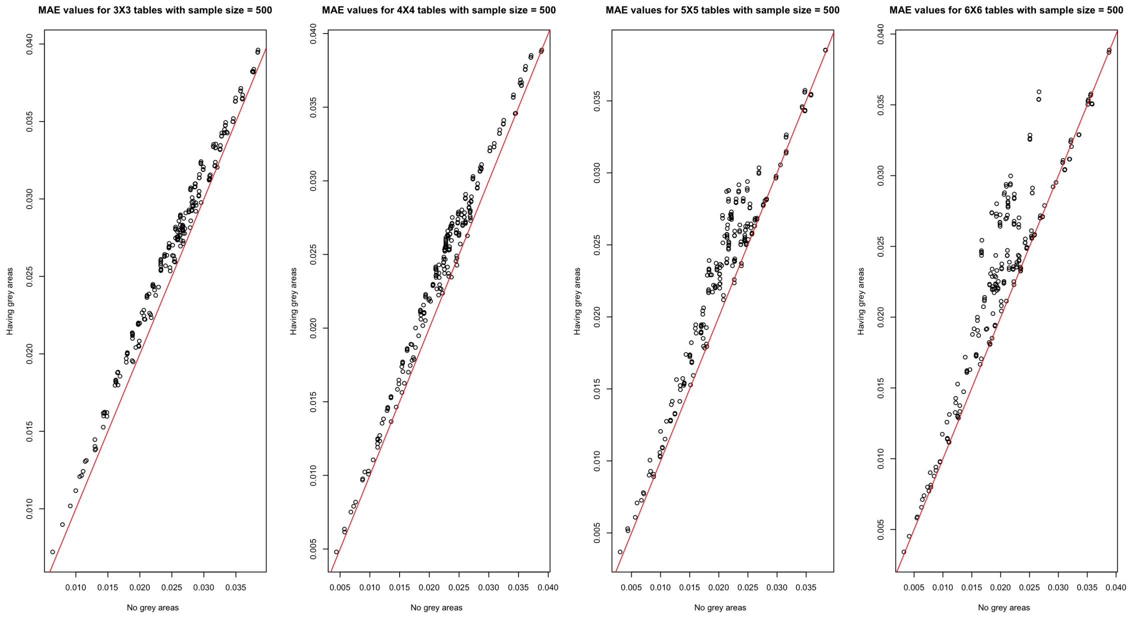

Figure 12: The comparison between two simulations for the sample size of 500 with 3x3, 4x4, 5x5, and 6x6 table sizes.

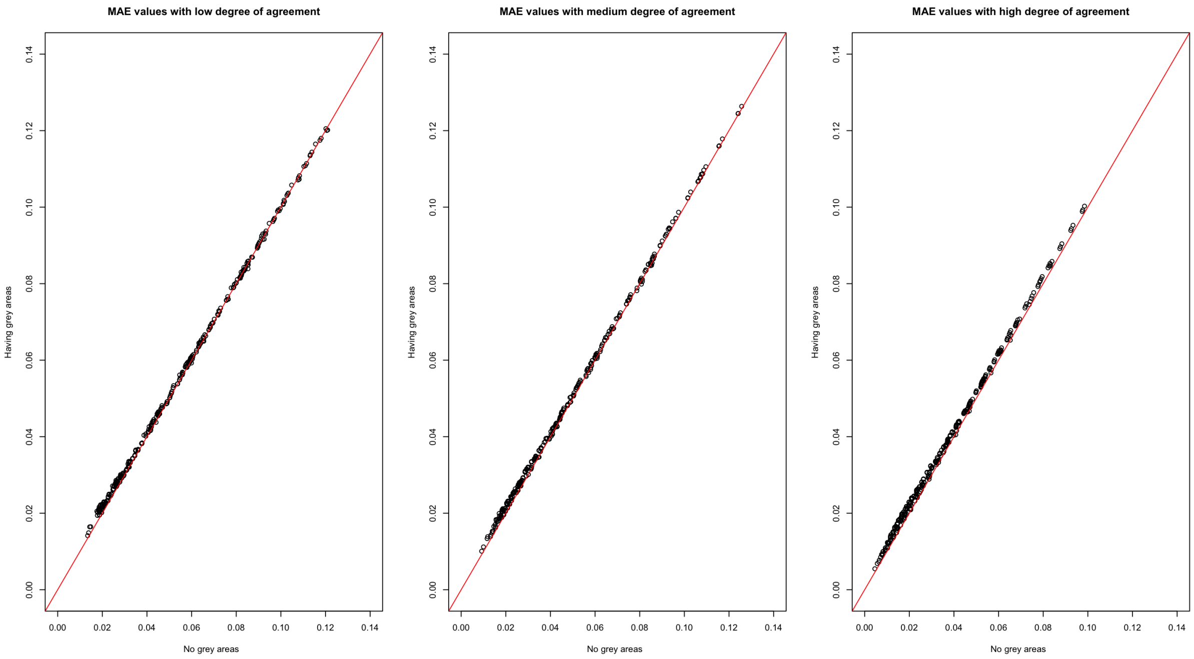

Figure 13: The comparison between two simulations with various degree of true agreements with low, medium and high degree of agreement.

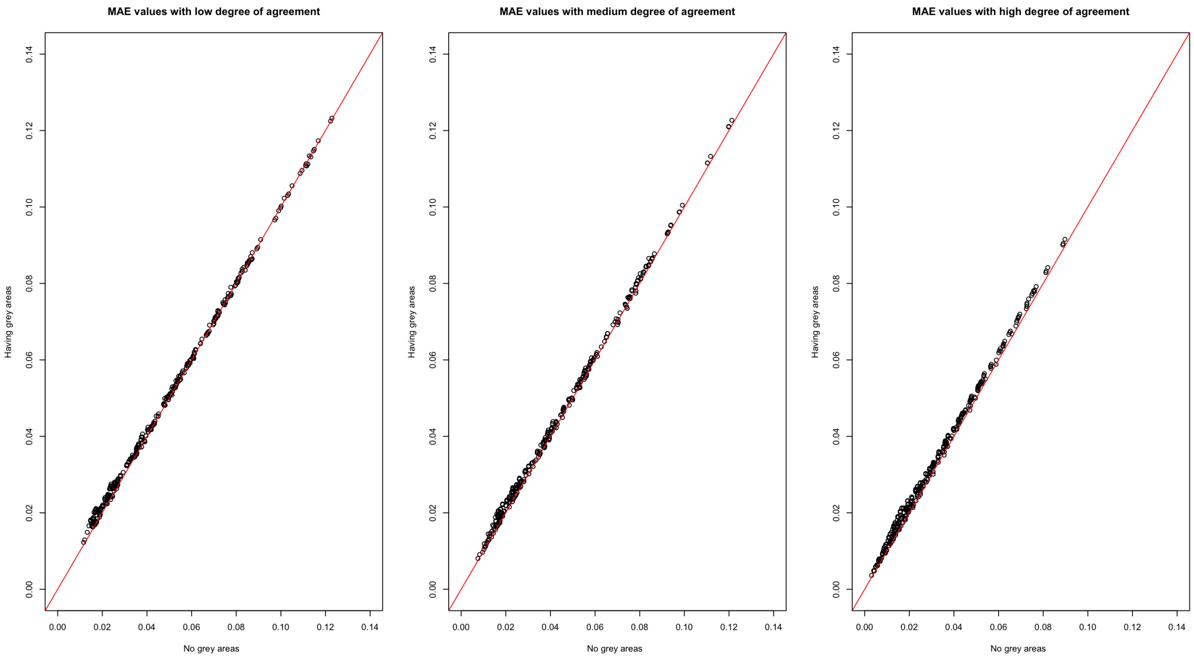

Figure 14: The comparison between two simulations with various degree of true agreements with low degree of agreement for tables with 3 to 6 categories.

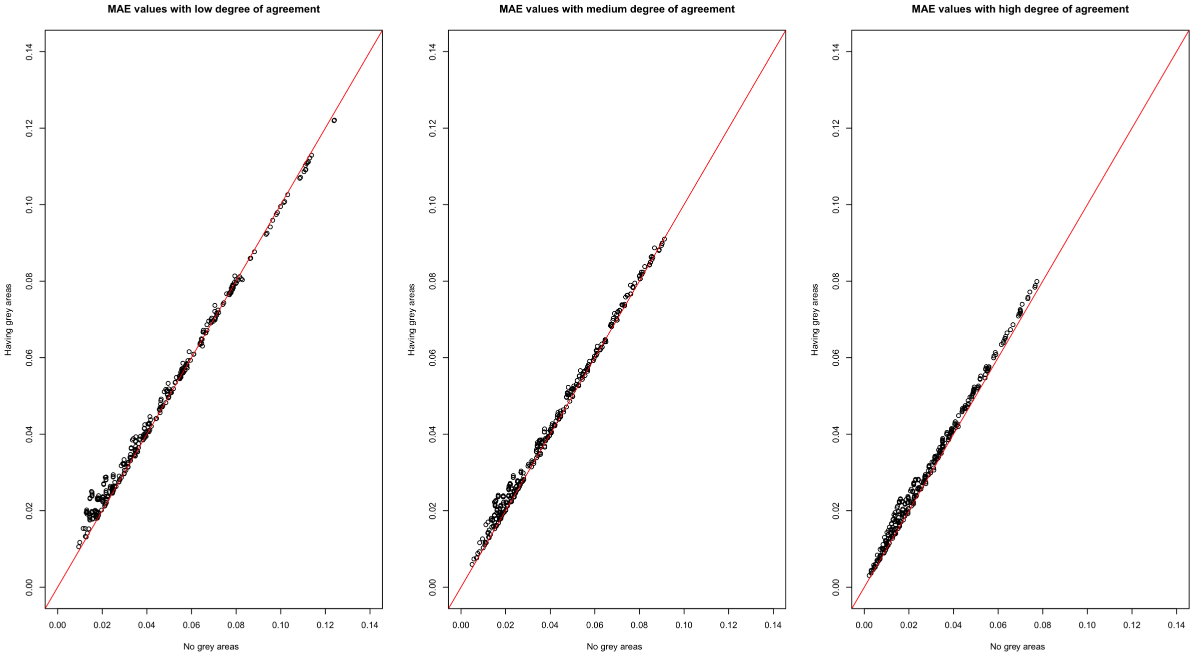

Figure 15: The comparison between two simulations with various degree of true agreements with medium degree of agreement for tables with 3 to 6 categories.
